# Conductive Atomic Force Microscope Study of Bipolar and Threshold Resistive Switching in 2D Hexagonal Boron Nitride Films

**DOI:** 10.1038/s41598-018-21138-x

**Published:** 2018-02-12

**Authors:** A. Ranjan, N. Raghavan, S. J. O’Shea, S. Mei, M. Bosman, K. Shubhakar, K. L. Pey

**Affiliations:** 1 Engineering Product Development, Singapore University of Technology and Design, 8 Somapah Road, 487372 Singapore; 2 Institute of Materials Research and Engineering, Agency for Science Technology and Research, 2 Fusionopolis Way, 138634 Singapore

## Abstract

This study investigates the resistive switching characteristics and underlying mechanism in 2D layered hexagonal boron nitride (h-BN) dielectric films using conductive atomic force microscopy. A combination of bipolar and threshold resistive switching is observed consistently on multi-layer h-BN/Cu stacks in the low power regime with current compliance (*I*_*comp*_) of less than 100 nA. Standard random telegraph noise signatures were observed in the low resistance state (LRS), similar to the trends in oxygen vacancy-based RRAM devices. While h-BN appears to be a good candidate in terms of switching performance and endurance, it performs poorly in terms of retention lifetime due to the self-recovery of LRS state (similar to recovery of soft breakdown in oxide-based dielectrics) that is consistently observed at all locations without requiring any change in the voltage polarity for *I*_*comp*_ ~1–100 nA.

## Introduction

Resistive random access memory (RRAM) technology is considered as a potential alternative to existing charge based memories^1^. The key attributes of RRAM include ultra-fast switching, ultra-low power consumption, multi-bit storage capability, superior endurance, high integration density, 3D stacking capabilities and simple fabrication requirements that are compatible with the traditional CMOS process^2,3^. The successful implementation and integration of RRAM technology with logic devices is showing great potential for use in neuromorphic computing applications^4^, physical unclonable function (PUF) circuits^5^ and true random number generators^6^.

The RRAM device is basically a nanoscale voltage/current controlled resistor, structurally consisting of either metal-insulator-metal (MIM) or metal-insulator-semiconductor (MIS) configuration, which can toggle between at least two different resistance states by formation of one or more conductive filaments (CF). The CF consists of either metal atoms/ions^7,8^ or vacancies in the insulator^9^ depending on the material configurations (both the anode and cathode electrode, oxide material) and current/voltage compliance conditions (i.e. breakdown hardness). A wide range of material systems have shown resistive switching including SiO_2_, HfO_2_, TiO_2_, Ta_2_O_5_, Al_2_O_3_ and Nb:SrTiO_3_^7–12^. However, the commercialization of the RRAM technology as a replacement to NAND Flash is limited due to high device-to-device as well as cycle-to-cycle variability, and other reliability issues that are still being addressed^13^.

Over the last decade, there has been an exponential growth in studies relating to graphene based nanoelectronic devices due to its unique properties such as ballistic transport at room temperature, mechanical flexibility, and favorable thermo-mechanical stability at high temperatures^14–16^. To realize the full potential of graphene technology, there is a search for suitable insulators which can be seamlessly integrated with graphene. Hexagonal boron nitride (h-BN) has emerged as a potential candidate for serving as an insulator on top of graphene since both are layered materials and have similar lattice parameters (lattice mismatch is only around 1.8%)^17^, which enables integration to form 2D heterostructures. h-BN is a large band gap material (~5.97 eV)^18^ with excellent chemical stability over wide temperature ranges^19^ and large thermal conductivity^20^. Additionally, h-BN has demonstrated high Young’s modulus of ~503 Nm^−1^ and breaking strength of ~15.7 Nm^−1^, which makes it suitable for flexible electronics applications^21^.

It is necessary to study the defect generation mechanism and switching mechanism of h-BN on graphene before the system can be used as 2D dielectric material for logic and memory devices. Recently, there have been a few studies which have probed the critical field strength of h-BN (~4–12 MV cm^−1^)^22,23^ and have reported some preliminary understanding of the mechanism of dielectric breakdown in h-BN. These studies point to a possible removal of material during hard breakdown and also suggest that breakdown is a layer-by-layer process for h-BN^24^. Additionally, there are a few reports investigating resistive switching in multi-layer h-BN at the device level using compliance currents in the μA-mA range, where the CF is inferred to be metallic in nature due to Ag, Ti or Cu migration from the electrodes^25–27^. However, limited understanding is available on the role played by intrinsic defects in h-BN on the resistive switching characteristics in the ultra-low power regime. The objective of this study is to focus on this regime to characterize the h-BN dielectric and to use the conductive atomic force microscope (CAFM) tip as a localized electrode, thus eliminating the need for deposition of a top metal electrode. The use of CAFM enables us to probe with spatial resolution of ~10 nm–100 nm for clear signatures and trends of localized switching. The CAFM approach has been previously used to study resistive switching in many bulk oxide thin films, such as HfO_2_, TiO_2_, NiO etc.^28–30^. There are only a few studies undertaken in ultra-high vacuum (UHV)^31,32^ and hence most previous CAFM studies have an inherent uncertainty in the electrical measurements because of contamination at the tip-sample contact.

## Results and Discussion

**Figure 1:**
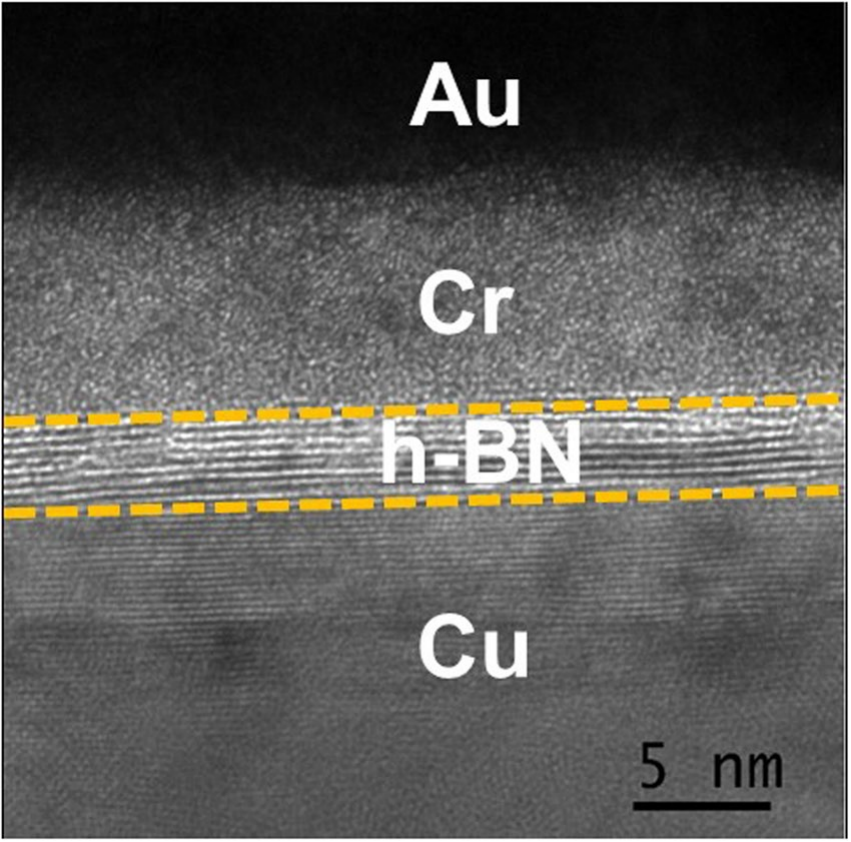
Physical Characterization: TEM micrograph of multi-layer h-BN grown on Cu substrate by chemical vapor deposition (CVD). The average number of h-BN layers varies between ~10–12. In preparing the sample for TEM analysis, the Au and Cr layers are deposited on top of h-BN/Cu.

In a CAFM experiment, an all-metal Platinum AFM cantilever is brought into mechanical contact with the h-BN surface (typical applied force ~20–40 nN) and the voltage bias on the tip is varied in a defined manner. Simultaneously, the current flow between the tip and sample is monitored. The current flow is always limited to a maximum defined current compliance (*I*_*comp*_), which is *I*_*comp*_ ~100 nA for all switching tests and *I*_*comp*_ ~10 µA for hard breakdown of the h-BN. The h-BN samples (from Graphene Supermarket^®^) are grown by chemical vapor deposition (CVD) directly on a ~20 μm thick Cu substrate. Figure 1 shows a TEM micrograph of the sample studied, with the layered growth of h-BN on Cu clearly visible. From TEM analysis, the average number of h-BN layers varies between ~10–12.

**Figure 2:**
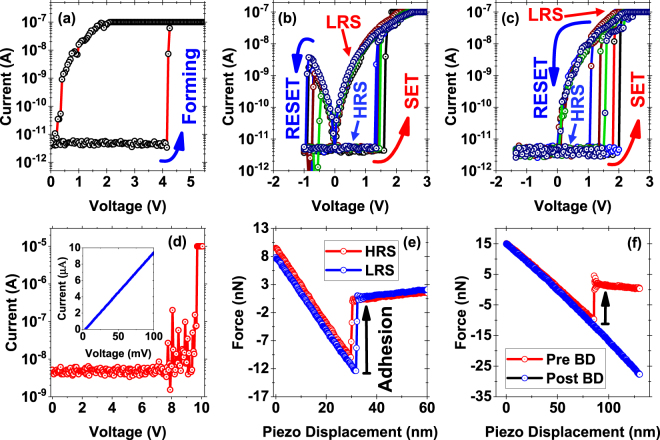
Resistive Switching Characteristics: Semi-logarithmic current-voltage (*I–V*) plots showing (**a**) forming and (**b**) bipolar resistive switching characteristics. (**c**) Threshold switching characteristics at the same location. *I*_*comp*_ is kept at ~100 nA for forming and all subsequent switching cycles. (**d**) Semi-logarithmic *I-V* plot showing the hard breakdown (HBD) characteristics when *I*_*comp*_ is set to ~10 μA. We do not observe any recovery here. Inset shows the post HBD characteristics obtained by applying a sweep voltage from 0–100 mV which shows an Ohmic behaviour with the resistance value equal to the ~10 kΩ protection resistance connected in series with the sample. (**e**) Adhesion force between h-BN and the Pt wire CAFM tip, measured when the tip is being retracted away from the h-BN surface. The HRS and LRS states refer to adhesion measurements taken under the resistive conditions as shown in (**b**) and (**c**). Negligible change is observed in adhesion force between the HRS and LRS state. (**f**) Similar adhesion measurements for the same location as in (**d**) prior and post HBD. Adhesion force after HBD has changed and is very high due to the formation of a metallic contact between the Pt tip and Cu substrate possibly via the rupturing of the h-BN layers.

The typical resistive switching characteristics of h-BN under the CAFM analysis are plotted in Fig. 2. Forming is needed to initiate the switching process (Fig. 2(a)), and subsequently a consistent bipolar switching is observed (Fig. 2(b)). Forming refers to the need to apply an initial high voltage to create a localized conduction path across the h-BN. In bipolar switching, when a positive ramp voltage is applied to the probed location, abrupt switching from high resistance state (HRS) to low resistance state (LRS) occurs near ~1.5 V. Similarly, when the polarity is reversed to negative bias, a sudden increase in the resistance is observed and the probed location changes its state from LRS to HRS. We note that the (*negative*) voltage required for RESET process is only a fraction of the (*positive*) SET voltage. Interestingly, for about half of the switching cycles, we consistently observed threshold switching (Fig. 2(c)), wherein the resistance value changed to HRS at low positive voltage while ramping down the voltage after SET; i.e. even before any bipolar switching could be attempted at negative bias.

When *I*_*comp*_ is increased to ~10 μA, we observe permanent dielectric breakdown, as shown in Fig. 2(d), following which no resistive switching is observed. The post-breakdown current-voltage (*I*–*V*) curve at the stressed location shows an Ohmic trend with resistance of ~10 kΩ (see inset of Fig. 2(d)), which is the value of the series protection resistance added into the experimental setup. To gain additional insights on the nature of the CF, we also measured the adhesion force of the AFM tip to the h-BN surface under different electrical stress conditions. For the virgin dielectric, the estimated adhesion force was 10–20 nN. At locations where we observe a consistent resistive switching with *I*_*comp*_ ~100 nA, the estimated adhesion force both in HRS and LRS also lies in the range of ~10–20 nN as shown in Fig. 2(e). The similar magnitude of adhesion force in LRS compared to HRS indicates that the h-BN underneath the AFM tip is physically intact and hence changes in the electrical properties must arise from stoichiometric and physical changes, e.g. an enhanced density of B vacancies (see below). There appears to be no externally induced nucleation of metallic filaments from either electrode. In direct contrast, the value of the adhesion force after the hard breakdown (*I*_*comp*_ ~10 µA) (Fig. 2(f)) increased dramatically. This increase in the adhesion force can be attributed to cold welded junctions formed by metal contacts under UHV conditions^33^, thus implying a metallic contact with the Pt CAFM tip due to either full removal (pitting) of h-BN or electromigration of Cu through h-BN leading to a direct contact. The formation of a Pt-Cu contact also accounts for the low Ohmic resistance.

**Figure 3:**
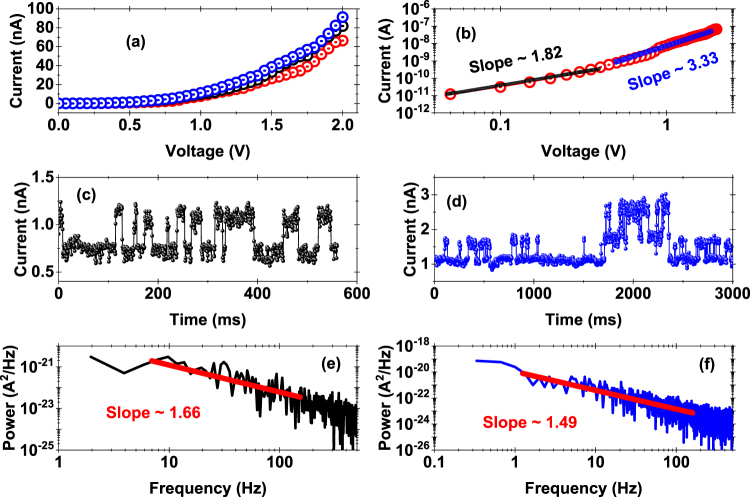
Analysis of Switching Mechanism. (**a**) Three typical *I–V* plots in LRS state. Non-linearity in the characteristic *I–V* curve was observed. (**b**) Plots of the *I–V* characteristics of (**a**) on a log-log scale clearly showing two different slopes over the voltage range of interest. (**c**) and (**d**) Random telegraph noise (RTN) trend observed in the LRS state. (**e**) and (**f**) Power spectrum density (PSD) plot of the RTN data of (**c**) and (**d**) respectively.

Returning to the case of *I*_*comp*_ ~100 nA, the *I-V* characteristics measured in LRS are shown in Fig. 3(a). The non-linear *I-V* profile implies that the conductive path is non-metallic and when plotted on a log-log scale, as shown in Fig. 3(b), we observe that the slope of the *I–V* curve can be split into two regimes. Initially, when the applied voltage is small (<0.5 V), the slope is ~1.82 ± 0.076 implying that the charge transport is governed by space charge limited conduction (SCLC)^34^. As the bias is increased (>0.5 V), the slope changes to *I ∝ V*^*m*^, where m = 3.33 ± 0.046. The conduction is this regime seems to be due to trap-assisted hopping of charge carriers through multiple layers of h-BN, and thus the overall conduction is dominated by trap-assisted SCLC^34–36^. These results show that the conduction in LRS is dominated by electrical traps/defects in the h-BN/Cu stack.

To further understand the charge transport and conduction mechanism, we used low voltage sensing to detect the noise patterns in the LRS state. Figure 3(c) and (d) shows examples of multi-level random telegraph noise (RTN) traces measured in the LRS state at the same location at a bias of 0.35 V and 0.40 V, respectively. The RTN signals clearly indicate defects undergoing stochastic carrier capture and emission^37–39^. The capture and emission time lies in the few milliseconds (ms) to 100 ms range. We analyzed the RTN traces in the frequency domain to obtain the low frequency response. Figure 3(e) and (f) show the power spectral density (PSD) of the two RTN traces in Fig. 3(c,d). Fitting the PSD versus frequency trend on a log-log plot yields a slope of 1.67 ± 0.06 and 1.49 ± 0.03, respectively, indicating that 2–3 defects are participating in the carrier capture and emission process at the given bias voltage^40,41^. The presence of low frequency Lorentzian RTN traces in the LRS state is additional proof that charge transport is governed by the defects/traps (e.g. Boron vacancy/ions)^37–41^ created during the SET process in the h-BN layers.

**Figure 4:**
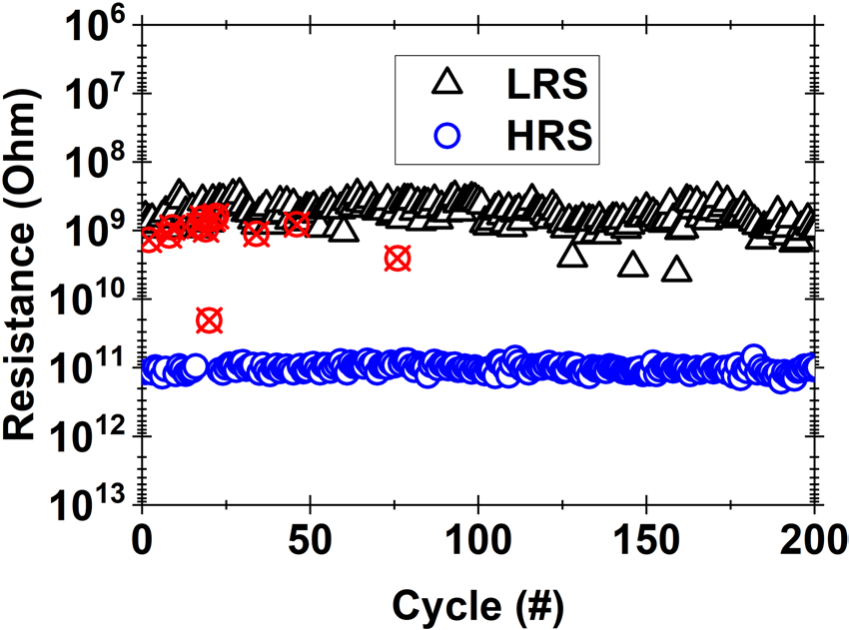
Endurance characteristics of resistive switching in h-BN/Cu stack across one of the probed locations at a reference read voltage of +0.5 V. “Switching soft error” data points are indicated by cross circles (red).

From a reliability perspective, the suitability of h-BN for non-volatile memory application is analyzed by means of endurance and retention tests. Bipolar switching tests were carried out at randomly chosen locations using the CAFM and we observed at least 100 consistent switching cycles in most cases. Figure 4 shows the resistance for one such location at a reference read voltage of +0.5 V for 200 switching cycles. The resistance memory window between HRS and LRS is maintained at around two orders of magnitude without any degradation. However, for a few switching cycles in the initial stage, we did observe a “switching soft error” where the probed location could not be immediately brought back to HRS. Interestingly, after a “switching soft error”, the HRS state could be recovered again in the next 1–2 switching cycles and resistive switching proceeded as per normal. We attribute this “switching soft error” to the kinetics of the local defects/vacancies forming the conduction path, which may not get passivated completely during the reset cycle. These local defects/vacancies can be passivated given more time over the subsequent switching cycles, thereby eventually reaching the HRS.

**Figure 5:**
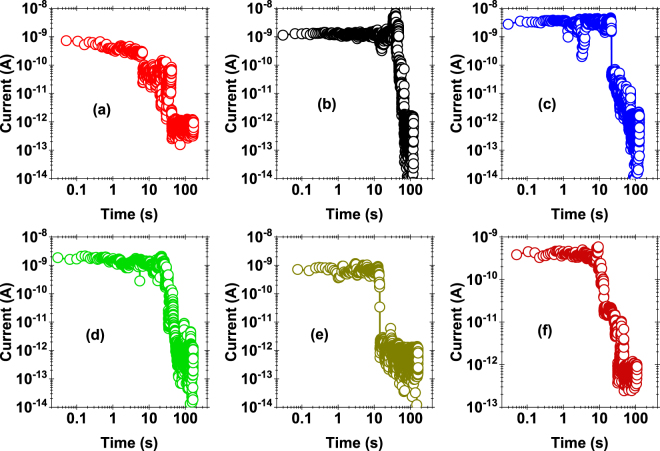
Retention characteristics of the h-BN/Cu stack across six different locations. After the probed locations are stressed to LRS, these locations are subjected to small read voltage lying in the range of 100 mV to 1 V. Self-recovery was consistently observed in time scales of ~10–100 sec and the probed location returned to the HRS state.

Retention is more of a concern in the LRS state. We used low bias sensing voltage, varying from 100 mV to 1 V, to monitor the retention characteristics once the probed location has been switched to the LRS state. Figure 5 shows the retention characteristics for six such probed locations. We consistently observed a drop in the value of the current from ~10^−9^ A to 10^−12^ A within a time scale ranging between 10 s–100 s, leading to a self-recovery of the dielectric breakdown in this soft breakdown (SBD) regime possibly due to concentration gradient driven back diffusion of ions from the electrode where they are stored. This suggests that while endurance performance is favorable, retention could be a bottleneck to the realization of h-BN RRAM, unless careful material engineering of the stack is performed. e.g. engineering a graphene interface between the electrode and h-BN, since graphene is known to serve as an effective barrier for ionic transport^26,27^.

A note of caution must be highlighted with regards to the conclusions drawn from the retention data because of thermal drift in the AFM experiments. That is, variation and changes in temperature will lead to thermal expansion of the components of the microscope and invariably cause the sample to move with respect to the tip over time. Relative movement of the tip over the surface of ~0.1 to 1 nm/s is not unusual at room temperature, although the use of a UHV environment does help minimize short term thermal fluctuation. Clearly, a large drift would be detrimental in conduction AFM measurements of highly localized events over long timescales, as in the case for the switching data presented above. Two observations can be made to give confidence that the retention loss phenomenon in Fig. 5 is intrinsic to the stack i.e. poor retention is a real occurrence and is not due to the tip drifting away from the switching site.

Firstly, the endurance tests (e.g. Fig. 4) were performed over similarly long timescales (up to ~10 minutes) and the switching between LRS and HRS states continued over the time of the experiment. Since a forming process is required to initiate switching at a new site on the surface, this observation indicates the tip remained over the switching site during endurance tests. This is compelling evidence that the tip does not drift appreciably during the experiments. One can assume retention tests show similar behavior.

A second, albeit less convincing, observation relates to consideration of the tip-sample contact mechanics. In the CAFM experiments, the applied force is reasonably high (~20–40 nN) and the tip is blunt (SEM measurement of Pt cantilever tips after use show a typical radius of curvature of ~100 nm, see supporting information - Fig. S1). This implies the friction force needed for the tip to slide is also high. That is, even though drift is occurring, in contact mode operation the tip must overcome the static friction forces and slide if it is to move away from a switching site. Sliding becomes more difficult with higher applied force and larger tip-sample contact area. A crude estimate indicates relative cantilever-sample displacements of a few nanometer (~1–4 nm) are required for slip to occur, showing that slip could occur after ~10–40 s using a reasonable drift rate of 0.1 nm/s under UHV conditions. In practice, the drift must be greater than ~1–4 nm to move away from the switching zone because the entire tip-sample contact area is electrically continuous and of finite value (a simple Hertz contact model gives the radius of the contact zone as ~3 nm). In summary, the contact mechanics estimates indicate that slipping and movement of the tip could occur in the ~10–100 second timescale, which is the same order as the retention measurements. Hence, from the contact mechanics alone, one cannot conclude the tip definitively remains over the switching zone. However, it is also clear that the calculations are extremely crude. Plastic deformation, surface roughness, and adhesion are some of the factors which influence the onset of sliding. A separate study relating drift, contact mechanics and conduction AFM is required to rigorously understand this issue.

**Figure 6:**
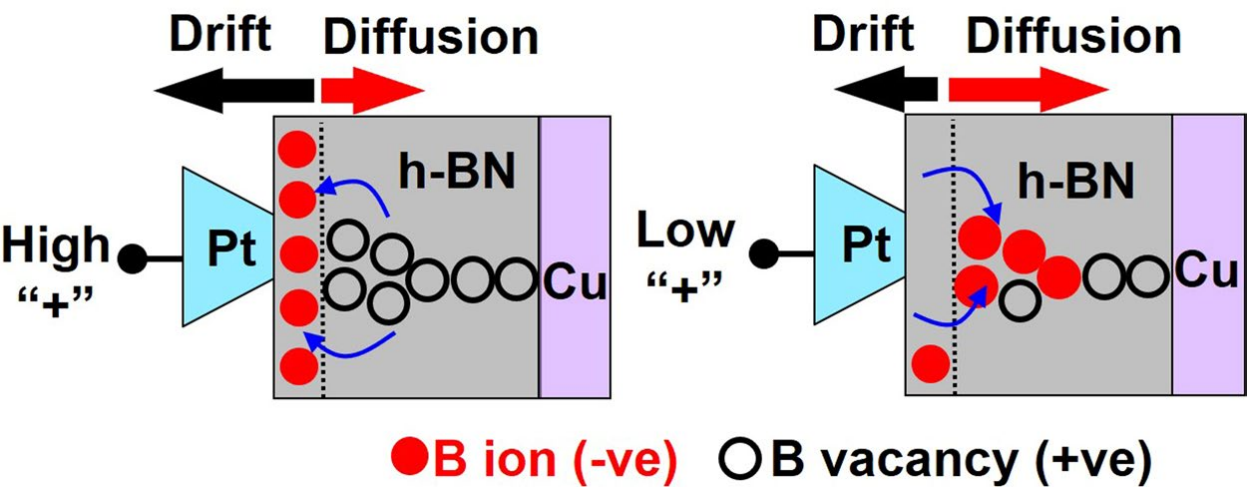
Schematics of the Resistive Switching Mechanism: Schematic showing the drift of boron ions (B^−^, solid balls) towards the Pt tip at high positive bias. At low bias, B^−^ ions diffuse back to annihilate the boron vacancy (open circles), reducing the overall conductivity of the h-BN/Cu stack. The directional movement of B^−^ ions is governed by the interplay between diffusion and drift. The length of arrows indicates qualitatively the relative magnitude of drift and diffusion flux of B^−^ ions.

*Ab initio* calculations have shown that formation and migration of boron vacancies in h-BN is energetically favorable compared to nitrogen vacancies^42^. Additionally, the low electron affinity of boron (compared to its first ionization energy) may energetically favor the formation of B^-^ ions. We propose here a simple model based on drift-diffusion principle (see Fig. 6) to explain threshold switching (self-recovery), poor retention and bipolar switching. For positive high voltage applied to the Pt wire tip, B^-^ ions drift towards the Pt tip forming B vacancies^42^ inside h-BN. These B^-^ ions tend to accumulate on the surface of h-BN close to the tip as a “sheet charge”. While B^-^ ions may diffuse back into the dielectric due to the high concentration gradient, the flux due to drift keeps most ions intact in the sheet charge during and just after SET operation. In the backward voltage sweep using the positive voltage cycle, the drift effect tends to gradually reduce and the diffusion flux begins to dominate. This causes B^-^ ions to diffuse back to passivate the B vacancies in h-BN, leading to a drop in overall conductivity at the probed location, which explains threshold switching and the severe retention loss (Fig. 5). In bipolar switching, the boron vacancy filament created during the SET process is not passivated during the reducing sweep in positive bias. In this situation, a small to moderate negative bias helps accelerate the reverse diffusion of boron ions back into the h-BN stack to passivate the boron vacancy sites, thereby annihilating the boron vacancy filament and reverting to the HRS state.

**Figure 7:**
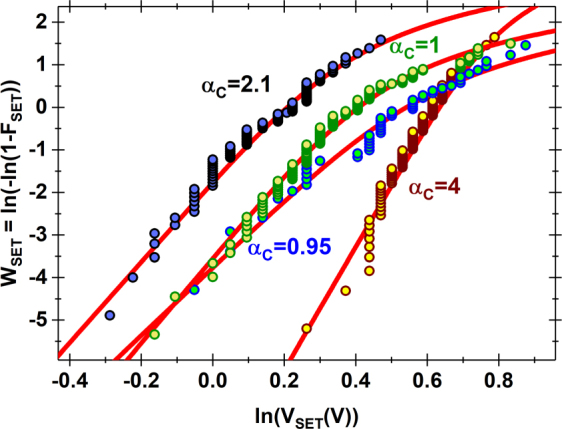
Clustering Model Fitting: Weibull plot of V_SET_ data at four different locations where bipolar switching was successfully demonstrated. The lines represent the clustering model best fit. Here, F_SET_ refers to the cumulative density function of the SET voltage and α_C_ is the fitted value of the clustering factor for each data plot. The symbol W_SET_ is the corresponding Weibit values for F_SET_.

Based on our proposed model that the switching phenomenon arises from the drift/diffusion of boron ions and vacancies, the statistical trend of *V*_*SET*_ was analyzed from several locations and plotted on a Weibull scale in Fig. 7. Here, *V*_*SET*_ is the (*positive*) SET voltage for bipolar resistive switching. It can be seen that the plotted data is not linear, suggesting that the Weibull model may not be directly applicable to this case. Alternatively, by applying the clustering model introduced by Wu *et al*.^43^ for dielectric breakdown, we observe a much better fitting of the same data sets. In the clustering model, the probability at a given voltage (*V*) is given by,

1 $${F}_{CLUS}=1-{(1+\frac{1}{{\alpha }_{C}}\cdot {(\frac{V}{\eta })}^{\beta })}^{-{\alpha }_{C}}$$

where *β*, *η* and *α*_*C*_ are the shape factor, mean transition voltage and clustering factor respectively. The lower the value of *α*_*C*_, the larger is the clustering effect. In the absence of any clustering (i.e. for a spatially random defect generation process), the typical value of *α*_*C*_ is more than 3. In our fitting results, we observe values of the cluster factor, from 0.5–4. The locations where *α*_*C*_ was on the low side (*α*_*C*_ < 3) may correspond to those where the previous reset was ineffective or “gradual” possibly implying that the presence of a few residual vacancy/ions from the previous switching cycle induces a non-random nature of defect generation and filament evolution for the next switching cycle. Similar phenomenon has been observed in the case of high-κ dielectrics^44^ where clustering has been attributed to lower activation energy for defect generation in the vicinity of an existing defect and higher binding energy for multi-vacancy defect/vacancy configurations^45–47^. Of the various locations where CAFM-based switching was observed on the blanket h-BN film, the predominance of low values of *α*_*C*_ suggests that filament evolution in h-BN is likely to show directional growth and not random regeneration of defects in the ruptured region for reconnectivity.

## Conclusion

In this study, we used the CAFM as a nanoscale tool to investigate the intrinsic switching mechanism in hexagonal boron nitride on copper in the ultra-low power regime. The stochastic analysis of switching trends show striking resemblance to the SET distributions in high-κ dielectric films, such as hafnium oxide. Evidence has been presented in favor of the reversible ionic transport of boron ions (B^−^) assisted by diffusion and drift resulting in bipolar and threshold switching. From a reliability viewpoint, while the endurance trends look promising, retention loss is a serious issue to be addressed due to self-back-diffusion of the boron ions. Further work is needed to understand how the bipolar and threshold switching events could be discretely controlled and how the retention loss could be prevented or improved, e.g. by inclusion of diffusion barrier layers such as graphene on top of h-BN.

## Experimental Section

Samples tested consist of commercially available h-BN (from Graphene Supermarket^®^) grown by chemical vapor deposition (CVD) directly on a ~20 μm thick Cu substrate. The samples were used as-received. The electrical measurements have been performed on this h-BN/Cu stack with an atomic force microscope (RHK Technology^®^) operating at ultra-high vacuum (UHV) (~10^−11^ mbar) conditions. All-platinum wire cantilevers (Rocky Mountain Nano Technology^®^, Model: RMN-12PT300B, spring constant = 0.8 Nm^−1^ ) were used in AFM contact mode for electrical measurements. After insertion into the vacuum chamber, tips were cleaned *in-situ* for ~3–4 minutes using Ar ion milling at ~1.2 keV. For all switching and noise measurements, the CAFM tip was always in physical contact with h-BN surface at a net applied force of ~20–40 nN. All tests were performed at room temperature.

A Keithley^®^ semiconductor characterization system (SCS 4200) was externally connected to the AFM to both measure the current from and apply voltage bias to the CAFM tip. The Cu substrate is grounded and a ~10 kΩ resistor is placed in series to protect against high current flow. The base level of noise for the measurement setup depends on the range of current selected in the Keithley^®^ SCS 4200 unit. The base level noise in current was 2 pA, 40 pA, 200 pA and 2 nA for 10 nA, 100 nA, 1 μA and 10 μA current ranges, respectively. An important feature of the Keithley^®^ SCS 4200 is that the compliance current can be precisely limited as the voltage is ramped, allowing us to probe the switching phenomena for a wide range of resistivity.

## Electronic supplementary material

Supplementary information
