# Supplementary material for: Conductive Atomic Force Microscope Study of Bipolar and Threshold Resistive Switching in 2D Hexagonal Boron Nitride Films
Source: Sci Rep. 2018 Feb 12;8:2854. doi: 10.1038/s41598-018-21138-x (PMC5809508; doi:10.1038/s41598-018-21138-x)
Supplement: Supplementary file 1 — Supplementary information [file 41598_2018_21138_MOESM1_ESM.pdf]

Supporting Information

Conductive Atomic Force Microscope Study of Bipolar and Threshold Resistive Switching in 2D Hexagonal Boron Nitride Films

A. Ranjan[1,2], N. Raghavan[1], S.J. O'Shea[2], S. Mei[1,2], M. Bosman[2], K. Shubhakar[1] and K.L. Pey[1]

[1]Engineering Product Development, Singapore University of Technology and Design, 8 Somapah Road, Singapore – 487372.

[2]Institute of Materials Research and Engineering, Agency for Science Technology and Research, 2 Fusionopolis Way, Singapore – 138634.

General Approach to Thermal Drift Estimation

In the estimation of drift, we assume that the friction force follows Amonton's Law and is proportional to μF_n, where F_n is the applied force and μ is the coefficient of friction. Under no-slip conditions, the lateral force (F_L) acting on the tip is $k_L \Delta x$, where Δx is the relative distance moved by the cantilever with respect to the surface and k_L is the relevant spring constant in the x direction. The spring constant in the lateral directions for a rectangular cantilever can be estimated using analytical expressions [1]. Using the manufacturer dimensions for the cantilever and taking 168 GPa and 61 GPa as the Pt elastic and shear modulus respectively, we find k_L~1-5 N/m. The tip will slip when the lateral force reaches μF_n i.e. $F_L = \mu F_n$ or equivalently $\Delta x = \mu F_n / k_L$ [2]. Hence, assuming a reasonable friction coefficient of μ=0.1 and taking F_n~40 nN, the tip will slip at Δx~1 to 4 nm. If the AFM drift rate is 0.1nm/s, these values of Δx correspond to times of 10-40 s.

[1] O'Shea, S.J.; Welland, M. E.; Wong, T.M.H.; Influence of frictional forces on atomic force microscope images *Ultramicroscopy* **1993**, 52, 54-64

[2] Tang, X.S.; Loke, Y.C.; Lu, P.; Sinha, S.K.; O'Shea, S.J.; Friction measurement on free standing plates using atomic force microscopy *Review of Scientific Instruments* **2013**, 84, 013702

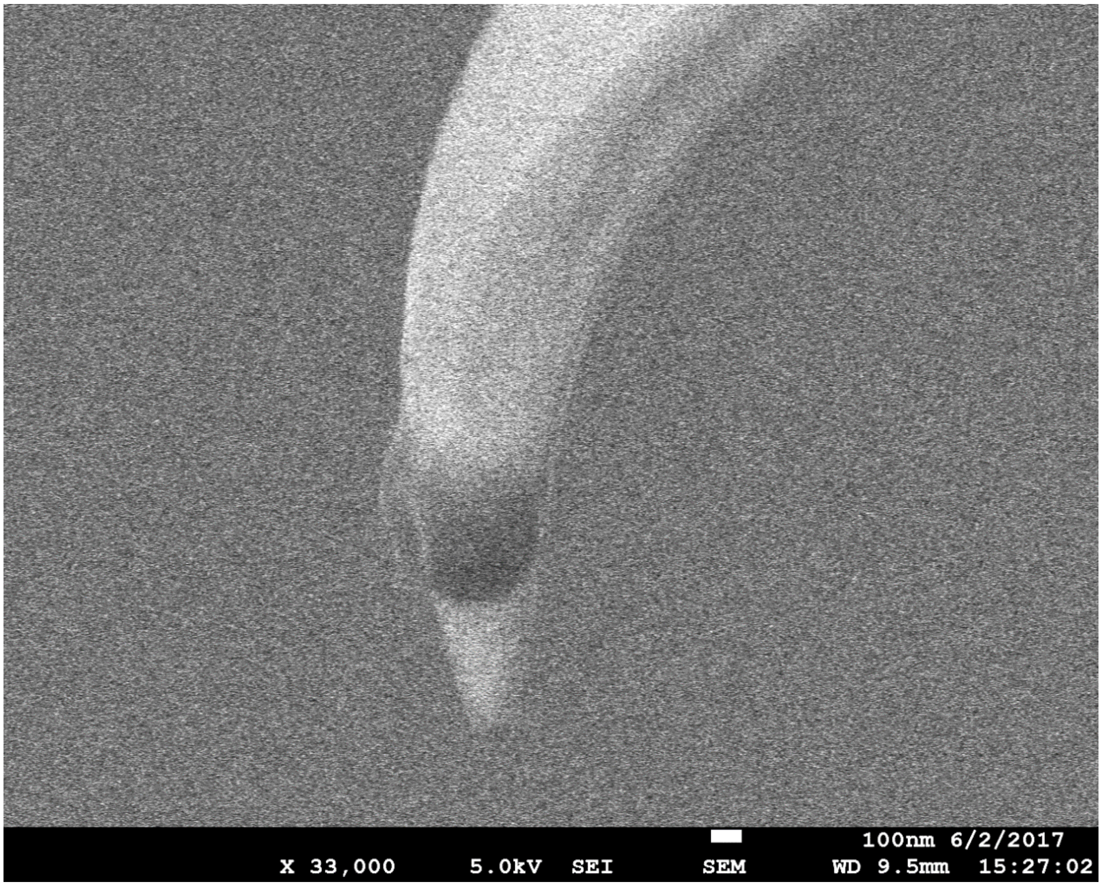

Figure S1 – *SEM image of platinum wire tip after electrical measurements are performed.*
